# Consumption of *Limosilactobacillus fermentum* Inhibits Corneal Damage and Inflammation in Dry Eye Disease Mouse Model through Regulating the Gut Microbiome

**DOI:** 10.3390/ijms25063528

**Published:** 2024-03-20

**Authors:** Kippeum Lee, Hyeonjun Gwon, Jae Jung Shim, Joo Yun Kim, Jae Hwan Lee

**Affiliations:** R&BD Center, Hy Co., Ltd., 22 Giheungdanji-ro 24 Beon-gil, Giheung-gu, Yongin-si 17086, Gyeonggi-do, Republic of Korea; joy4917@hanmail.net (K.L.); hjgwon@hy.co.kr (H.G.); jjshim@hy.co.kr (J.J.S.); jaehwan@hy.co.kr (J.H.L.)

**Keywords:** corneal disease, dry eye, *Limosilactobacillus fermentum*, gut–eye axis microbiome, pro-inflammatory regulation, tight junction

## Abstract

The present study investigated the effect of orally administered *Limosilactobacillus fermentum* HY7302 (HY7302) on the relationship between ocular tissue and the microbiome in a corneal injury dry eye mouse model. Specifically, 0.1% benzalkonium chloride (BAC) was applied to the ocular surface for 14 days to induce corneal injury in male Balb/c mice. During the BAC treatment period, HY7302 (1 × 10^8^ CFU/kg/day or 1 × 10^9^ CFU/kg/day) or an omega-3 positive control (400 mg/kg/day) were administered orally (*n* = eight/group). To examine the signaling pathways affected by the HY7302 treatment, the in vitro effects of HY7302 on the tight junctions and the inflammatory response were investigated in the mouse colon epithelial cell line, CMT-93. BAC exposure decreased tear production, induced ocular inflammation and corneal epithelial detachment, and altered the gut microbiota. However, oral administration of HY7302 restored tear secretion and decreased corneal epithelial detachment in BAC-treated corneal injury mice. Further, HY7302 alleviated corneal inflammation via modulation of matrix metalloproteinase-9 (MMP-9) expression and affeted alterations in gut microbiota composition. These findings suggest that the gut–eye axis interaction between gut microbiota and corneal tissue affects disease severity in corneal injury, and that the alteration of the microbiota by HY7302 could improve eye health by regulating the inflammatory response.

## 1. Introduction

Dry eye (DE) is a multifactorial disease of the ocular surface caused by impairment of tear production and cornea damage, which affects 5–40% of adults over 40 years of age [1]. The prevalence of DE is steadily increasing, mainly due to the widespread use of electronic devices, and DE is one of three major eye diseases rapidly increasing in the elderly [2,3]. The primary symptoms of DEs are stiffness, vision blurring, eye fatigue, and eye congestion. Dysfunction of the tear film can cause damage to the cornea and other epithelial eye tissue, preventing them from functioning correctly [4]. Also, DE is closely related to inflammation-induced tear film and ocular surface damage [5]. DE can result in epithelial lesions or a local inflammation reaction, leading to a deterioration of the ocular surface defense mechanisms, dysfunction, and cellular degeneration of the conjunctiva or cornea tissue [6]. Therefore, it is meaningful to attempt to establish a therapeutic approach to DE treatments. Despite recent attempts through DE pharmacological treatment studies, insufficient therapeutic efficacy and low ocular viability have been reported as major problems [3]. Other approaches, namely artificial tears containing hyaluronic acid or cyclosporine A that are used to treat DE, provide only temporary symptomatic relief [7]. Long-term application of these agents can have adverse effects, such as corneal and ocular hypertension, infection, and inflammation [8]. Benzalkonium chloride (BAC) is the most commonly used preservative in topical artificial tear products. Although BAC products have an amphiphilic, highly water-soluble characteristics, and antimicrobial effects, side effects can occur during long-term use, including DEs and ocular inflammation [9,10]. Even recent studies have reported that BAC can reach the posterior eye and optic nerve [11,12].

Cornea injury is a representative feature of dry eyes and is characterized by ocular surface inflammation and destruction of the tear film due to the up-regulation of inflammatory cytokines [13]. Also, ocular inflammation is considered to be one of the hallmarks of DEs. The production of pro-inflammatory triggers, including interleukin-20 (IL-20) and tumor necrosis factor-α, onto the ocular epithelial surface and the subsequent damage causes tear film dysfunction and, ultimately, DEs [14]. Metalloproteinases (MMPs) are positively associated with the severity of inflammation in the conjunctiva tissue, and recent studies have identified MMPs as a potential therapeutic target for DE [15]. MMPs also disrupt tight junctions essential for maintenance of the corneal barrier. In DE, matrix metalloproteinase-9 (MMP-9) levels are increased in the tears and ocular epithelial surface [16]. Exposure of the cornea to desiccating stress increases corneal epithelial permeability, which is regulated in part by increased MMP-9 levels [17,18,19]. Also, pro-inflammatory cytokine IL-20 is involved in the pathogenesis of inflammatory DE disease. A recent study identified that the circulating level is significantly increased in DE patient tears and corneas, and in induced DE models [20]. 

The gut microbiota regulates host physiological processes via strengthening gut tight junctions and regulating the intestinal epithelium, with improved function associated with increased microbial diversity [21]. The composition and activity of the gut microbiota affect host health by causing changes in metabolic activity or changes in local distribution [22]. For instance, certain symbiotic bacteria, such as Bifidobacterium, can prevent colonization of pathogenic bacteria by reducing the intestinal pH [23]. Also, with the recent enhanced understanding of the important role of the gut microbiome, there are researches on the relationship of host inflammation response and the pathogenesis of ocular diseases [24]. Recent studies have reported an association between ocular diseases and the microbiota profile of the host intestine. This is called the ‘gut–eye axis’, which indicates that changes in the gut microbiome alter host immunity, with a consequential influence on ocular health and disease [25,26]. Furthermore, the gut microbiome is associated with a myriad of pathophysiological processes in the host, especially chronic inflammatory diseases [27,28,29,30]. Therefore, probiotics have recently attracted attention as a potential dietary supplement to prevent inflammation. Some of the major mechanisms of immune system-related benefits of probiotics studied in vitro and in vivo studies are the enhancement of the epithelial barrier and the regulation of inflammatory cytokine production. Indeed, numerous studies have reported that *Lactobacillus* and *Bifidobacterium* have the ability to accelerate the anti-inflammatory process and reduce the production of the pro-inflammatory cytokines, IL-1b or IL-6.

Bacteria belonging to the phylum Firmicutes are some of the most important probiotic bacteria in the gut microbiome. Firmicutes are widely distributed in nature, and include *Limosilactobacillus fermentum*, *Limosilactobacillus reuteri*, *Lactobacillus acidophilus*, *Lacticaseibacillus casei*, and *Lactobacillus delbrueckii* subsp. *bulgaricus.* In particular, *Limosilactobacillus fermentum* (*L. fermentum*) is an obligately heterofermentative microbiota that ferments carbohydrates to produce lactic acid, ethanol, acetic acid, and carbon dioxide [31]. Recent studies have reported beneficial effects of *L. fermentum* in regard to obesity, cardiovascular disease, metabolic mellitus, and gastrointestinal barrier dysfunction [32,33,34,35]. It is also known, through animal experiments, that *L. fermentum* acts as an antimicrobial and antioxidant modulator [36]. In our previous study, we identified that oral administration of *Limosilactobacillus fermentum* HY7302 (HY7302) improved DE symptoms in a mouse model [37]. However, the molecular mechanisms of the effects of probiotic intake on ocular tissues have not yet been elucidated. Therefore, in this study, to understand the efficacy and molecular mechanisms of probiotics on DE-induced corneal damage, we determined the signaling regulation of inflammatory and apoptotic factors in ocular tissues after HY7302 intake in BAC-induced DE mice. To understand the mechanisms behind the therapeutic effects of HY7302 in DEs, it is essential to delineate the role of the gut–eye axis and microbiome in regulating DE pathology. Thus, in this study, we investigated the correlation effect of HY7302 probiotics with the intestinal microbiome of mice with corneal damage.

## 2. Results

### 2.1. HY7302 Ameliorated BAC-Induced DE Symptoms

**Figure 1:**
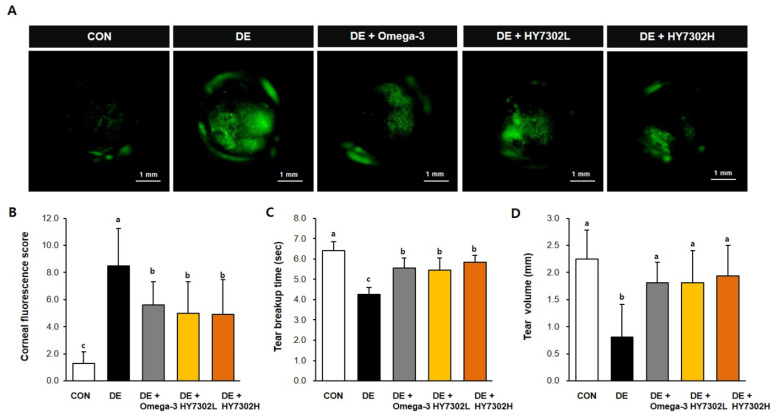
Effect of HY7302 on corneal fluorescein score, tear break-up time, and tear volume. (**A**,**B**) Corneal fluorescein images and corneal fluorescein sodium staining (CFS) scores in DE mice. (**C**) Tear break-up time as detected with commercial fluorescein strips. (**D**) Tear volume as measured by Schirmer’s test. CON, non-DE control group; DE, topical 0.1% BAC; DE + omega-3, topical 0.1% BAC + oral omega-3 (400 mg/kg/day); DE + HY7302L, topical 0.1% BAC + oral HY7302 (10^8^ CFU/kg/day); DE + HY7302H, topical 0.1% BAC + oral HY7302 (10^9^ CFU/kg/day). Data are expressed as the mean ± SD (*n* = 6). Values with different letters are significantly different; *p* < 0.05 (a > b > c).

To examine the differences in the physiological characteristics between the control and DE mice, the eyes of the mice in the DE groups were exposed to benzalkonium chloride (BAC) twice daily for 14 days. During the same period, the mice received once daily oral administration of the vehicle control, low-concentration HY7302 (1 × 10^8^ CFU/kg/day), high-concentration HY7302 (1 × 10^9^ CFU/kg/day), or omega-3 (400 mg/kg/day) as a positive control. To evaluate corneal epitheliopathy following oral administration of HY7302, corneal fluorescein staining was conducted, with representative images shown in Figure 1A. Significant differences in the CFS scores were present between the non-DE control (CON) and DE groups. The CFS scores in the HY7302 groups were similar to that of the omega-3 group, showing a significant decrease compared with the DE groups (Figure 1B). Furthermore, BAC treatment significantly decreased the TBUT by 36% and tear volume (TV) by 66% relative to the CON. Treatment with 1 × 10^8^ CFU/kg/day HY7302 recovered the TBUT to 128% and TV to 223% relative to the vehicle-treated DE group. Treatment with 1 × 10^9^ CFU/kg/day HY7302 increased the TBUT to 137% and TV to 238% relative to the vehicle-treated DE group (Figure 1C,D). The positive control, omega-3-treated group showed an increased TBUT of 130% and TV of 228% relative to the DE groups.

### 2.2. HY7302 Improved Corneal Epithelial Damage in Mice with BAC-Induced Cornea Damage

**Figure 2:**
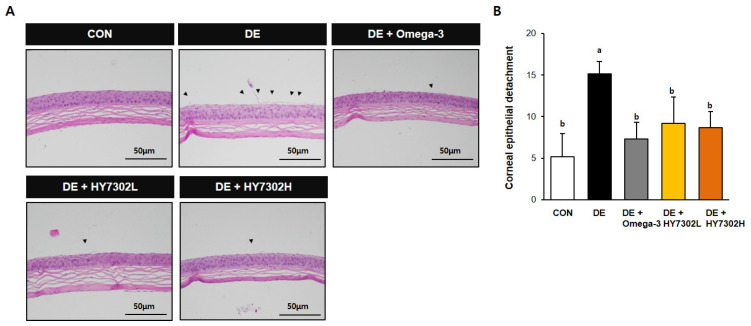
Effect of HY7302 on corneal epithelial detachment. (**A**) Representative hematoxylin and eosin images of DE mouse corneas. Arrows indicate detaching damaged apical tissue. (**B**) Quantification of corneal epithelial detachment, expressed as mean ± SD (N = four eyes/group). Values with different letters are significantly different; *p* < 0.05 (a > b).

Following 14 days of topical ocular BAC treatment, detached epithelial flaps and damaged corneal basal cells were present in the H&E images. However, the epithelial morphology of HY7302- and omega-3-treated DE mice was improved compared with the untreated DE group (Figure 2A). The corneal epithelial tissue dissections were counted in each group, with a 60% decrease in dissections in HY7302L-treated animals and a 57% decrease in HY7302H-treated animals relative to the DE groups (Figure 2B). Also, the omega-3 group showed a 57% decrease in the dissection score compared to the DE groups.

### 2.3. HY7302 Regulation of Pro-Inflammatory Factors in Mice with BAC-Induced Cornea Damage

**Figure 3:**
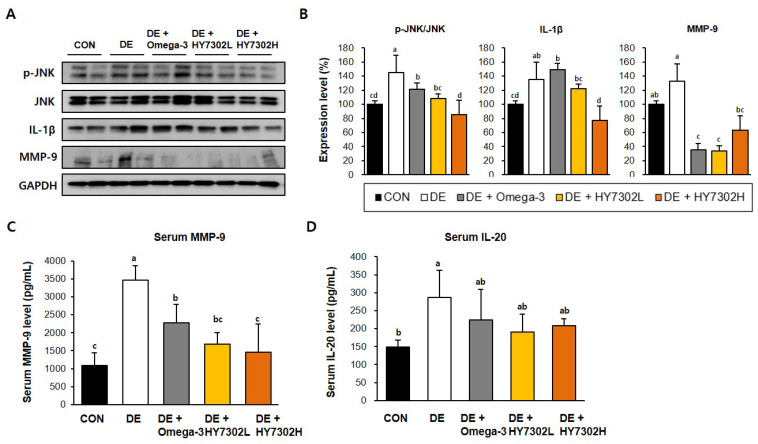
Effect of HY7302 on serum and corneal tissue pro-inflammatory factor levels in BAC-induced DE mice. (**A**) Western blot images of extracellular signal-regulated kinase (ERK), phospho-ERK (p-ERK), c-Jun N-terminal kinase (JNK), phospho-JNK (p-JNK), matrix metalloproteinase-9 (MMP-9), inteleukin-1 beta (IL-1β), and glyceraldehyde 3-phosphate dehydrogenase (GAPDH). (**B**) Quantification of phosphoprotein ratios or protein levels relative to CON. Serum concentrations of (**C**) matrix metalloproteinase-9 (MMP-9) and (**D**) interleukin-20 (IL-20) were measured using ELISA. Data are expressed as the mean ± SD (*n* = 6). Values with different letters are significantly different; *p* < 0.05 (a > b > c > d).

Pro-inflammatory regulators initiate immune activation by the corneal epithelium, which is a defining characteristic of DEs. Recent studies have identified up-regulation of MMPs as a pathologic change in DE patients and in animal models [38,39]. We measured the pro-inflammatory protein levels in corneal tissue and serum to delineate the molecular mechanisms by which HY7302 alleviated DE (Figure 3A,B). The p-ERK/ERK ratio, p-JNK/JNK ratio, IL-1β level, and MMP-9 level were significantly increased in the corneal tissue of DE mice. HY7302 administration decreased the p-ERK/ERK ratio, p-JNK/JNK ratio, and IL-1β level relative to the untreated DE group in a dose-dependent manner. The MMP-9 levels were also decreased in HY7302-treated DE mice. By contrast, omega-3 treatment did not affect the p-JNK/JNK ratio or IL-1β level, but decreased the MMP-9 level. Serum MMP-9 levels were 3.2-fold higher in DE mice than in CON mice (Figure 3C,D). Serum MMP-9 levels were significantly decreased, by 0.48-fold, in HY7302L-treated mice and 0.42-fold in HY7302H-treated mice relative to DE mice, which was more effective than the decrease by 0.65-fold in the omega-3-treated mice. Lastly, serum IL-20 was significantly decreased, by 0.84-fold, in HY7302H-treaed mice relative to DE mice, which was more effective than the omega-3 treatment.

### 2.4. HY7302 Decreased Activation of Ocular Apoptotic Pathways in Mice with BAC-Induced Cornea Damage

**Figure 4:**
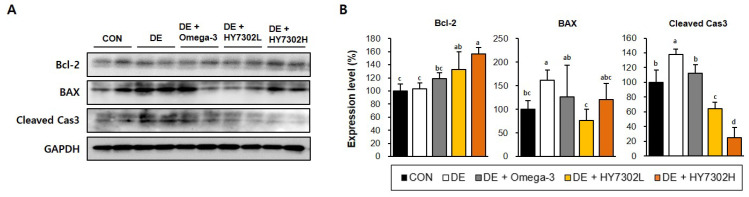
Effect of HY7302 on apoptotic factor levels in corneal tissue of BAC-induced DE mice. (**A**) Western blot images of B-cell lymphoma 2 (Bcl-2), Bcl-2-associated X protein (BAX), cleaved caspase-3 (Cleaved Cas3), and glyceraldehyde 3-phosphate dehydrogenase (GAPDH), and (**B**) quantification of protein levels relative to the CON. Statistical significance was determined using a one-way ANOVA, followed by Duncan’s test (*n* = 4). Values with different letters are significantly different; *p* < 0.05 (a > b > c > d).

To evaluate the effect of the HY7302 probiotic treatment on ocular surface apoptosis, the protein levels of the apoptotic factors were measured using Western blot analysis. The BAX and cleaved caspase-3 levels were significantly increased in the corneal tissue of DE mice relative to the CON mice. But the Bcl-2 expression level in DE mice showed no significant difference compared with those of the CON mice in this study. Treatment with HY7302 reversed the effects of DE on the Blc-2 and cleaved caspase-3 levels in a dose-dependent manner. Especially, HY7302H administration increased 51.9% of Bcl-2 and decreased 25.2% of BAX, compared with DE respectively. However, the BAX levels were significantly decreased by the HY7302L treatment, but non-significantly decreased by the HY7302H treatment. Contrastingly, treatment with omega-3, which was used as a positive control, did not affect the apoptotic factor levels in DE mice (Figure 4).

### 2.5. Effect of HY7302 Probiotics on Microbial Diversity and Composition in Mice with BAC-Induced Cornea Damage

**Figure 5:**
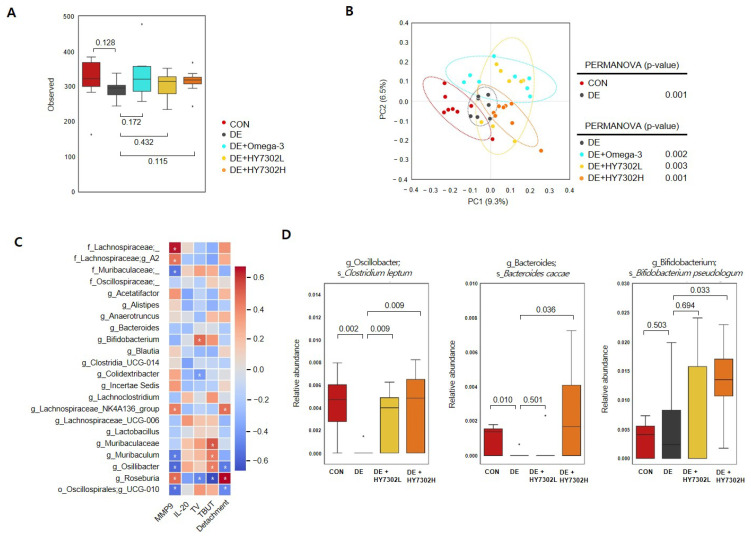
Effect of HY7302 on gut microbiota diversity, Spearman’s correlation, and relative species-level abundances in BAC-induced DE mice. (**A**) Box plots of microbial alpha diversity of each group was calculated using observed features. (**B**) PCoA plots of the bacterial community using unweighted UniFrac distance and PERMANOVA were used to measure the dissimilarity between groups. (**C**) Spearman’s correlation analysis between genus-level taxonomy profiles and DE indicators (serum MMP-9, serum IL-20, TV, TBUT, and corneal epithelial detachment). Regarding the heatmap, red squares indicate positive correlations and blue squares indicate negative correlations. (* *p* < 0.05). (**D**) Relative changes in abundance of microbiota species (*Clostridium leptum*, *Bacteroides caccae*, *Bifidobacterium pseudolongum*) at the baseline and after HY7302 treatment.

The composition of the gut microbiota was analyzed in each treatment group (Figure 5). The alpha diversity of the fecal microbiome community in the treatment groups (CON, DE, DE + omega-3, DE + HY7302L, DE + HY7302H) was characterized using the observed index (Figure 5A). There were no significant differences in microbial community diversity between the groups, according to the alpha diversity. However, the index data revealed that microbiome richness was modestly decreased in the DE groups relative to the CON group. Beta diversity was analyzed by calculating the unweighted UniFrac distance, to examine differences in microbial community composition and structure. Significant differences were detected between the CON mice and DE mice, and between DE + omega-3, DE + HY7302L, and DE + HY7302H mice relative to the DE mice (Figure 5B). This suggested that HY7302 could regulate the microbial community in BAC-induced DE mice. The Spearman correlation of microbiota taxonomic levels with in vivo experimental DE parameters, including serum MMP-9, serum IL-20, TV, TBUT, and corneal epithelial detachment damage in the DE, DE + HY7302L, and DE + HY7302H group was determined (Figure 5C). The relative Bifidobacterium abundance positively correlated with the TV (ρ = 0.48), while the Colidextribacter abundance negatively correlated with the TV (ρ = −0.41). Further, the Lachnospiraceae family (Lachnospiraceae A2, Lachnospiraceae NK4A136 group, Roseburia, and Blautia) abundance negatively correlated with the TV in the HY7302-treated groups. The TBUT index positively correlated with abundances of the family Muribaculaceae, genus Muribaculum, Oscillibacter, Oscillospirales UCG-010, and Bifidobacterium. In most taxonomic analyses, the TBUT index was similar to the TV. Corneal epithelial detachment was positively correlated with the abundance of Roseburia and Lachnospiraceae NK4A136, and negatively correlated with the abundance of Oscillibacter and Oscillospirales UCG-010. The serum MMP-9 levels positively correlated with the abundance of the family Lachnospiraceae, Lachnospiraceae A2, Lachnospiraceae NK4A136, Roseburia, and Colidextribacter, while the serum MMP-9 levels negatively correlated with the abundance of the family Muribaculaceae, Muribaculum, Oscillibacter, Oscillospirales UCG-010, Bifidobacterium, and Lactobacillus. Lastly, the relative microbiota abundance was evaluated at the species level. The relative abundance of *Clostridium leptum* and *Bacteroides caccae* were decreased in the DE group relative to the CON group. The abundance of *B. caccae* was significantly increased in the DE + HY7302H group relative to the DE group and was modestly increased in the DE + HY7302L group relative to the DE group. *C. leptum* (genus Oscillibacter) abundance was decreased in the DE group relative to the CON group, while *C. leptum* abundance was increased in the DE + HY7302L and DE + HY7302H groups relative to the DE group. Interestingly, HY7302H treatment significantly altered *B. pseudolongum* abundance compared with the CON and DE groups, which positively correlated with the TV. 

### 2.6. HY7302 Increased Transcript Levels of Tight Junction Components and Decreased Pro-Inflammatory Factor mRNA Levels in the Mouse CMT93 Cell Line

**Figure 6:**
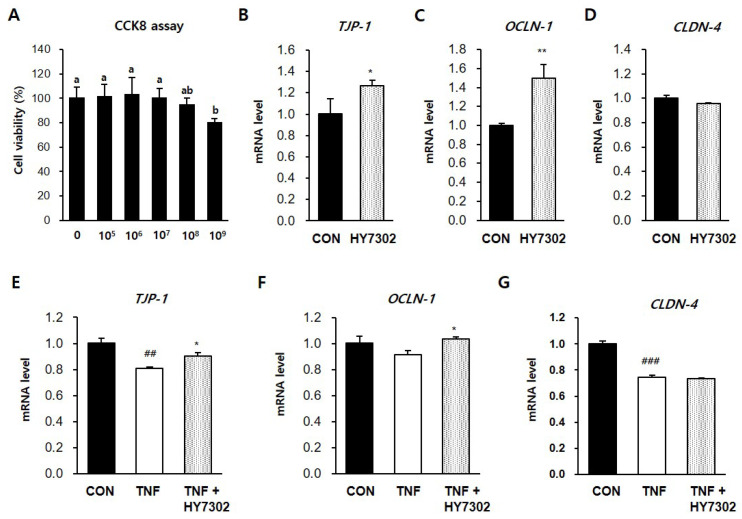
Effect of HY7302 on mRNA levels of tight junction components in TNFα-stimulated CMT93 cells. (**A**) Cell viability of CMT93 cells, following 24 h HY7302 treatment, was detected using a Cell Counting Kit-8 (CCK-8) assay. (**B**–**D**) mRNA levels of (**B**) tight junction protein-1 (TJP-1), (**C**) occuludin-1 (OCLD-1), and (**D**) claudin-4 (CLDN-4) in CMT93 cells treated with the vehicle (CON group) or 107 CFU/mL HY7302 (HY7302 group) were measured using quantitative-PCR analysis. (**E**–**G**) The mRNA levels of (**E**) TJP-1, (**F**) OCLD-1, and (**G**) CLDN-4 were measured using qPCR analysis in CMT93 cells treated with the vehicle (CON group), 100 ng/mL TNFα (TNF group), or 100 ng/mL TNFα + 10^6^ CFU/mL HY7302 (TNF + HY3702 group). Data are expressed as the mean ± SD (*n* = 4). CCK8 values with different letters are significantly different; *p* < 0.05 (a > b). ## *p* < 0.01 and ### *p* < 0.001 compared with C group. * *p* < 0.05 and ** *p* < 0.01 compared with DE group.

**Figure 7:**
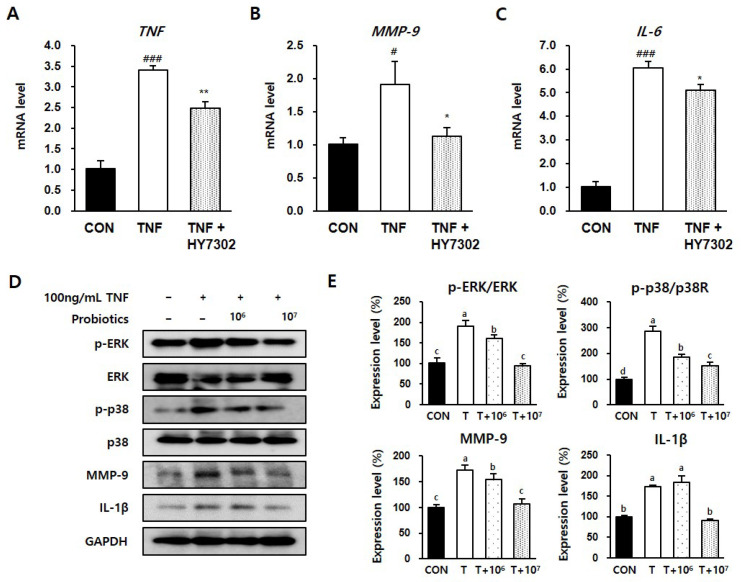
Effect of HY7302 on pro-inflammatory factors in TNFα-stimulated CMT93 cells. (**A**–**C**) The mRNA levels of (**A**) tumor necrosis factor (*TNF*), (**B**) matrix metalloproteinase-9 (*MMP-9*), and (**C**) inteleukin-6 (IL-6) were measured using qPCR analysis in CMT93 treated with the vehicle (CON group), 100 ng/mL TNFα (TNF group), or 100 ng/mL TNFα + 10^7^ CFU/mL HY7302 (TNF + HY7302 group). (**D**) Western blot images of extracellular signal-regulated kinase (ERK), phospho-ERK (p-ERK), p38 mitogen-activated protein kinases (p38), phospho-p38 (p-p38), matrix metalloproteinase-9 (MMP-9), inteleukin-1 beta (IL-1β), and glyceraldehyde 3-phosphate dehydrogenase (GAPDH), and (**E**) phosphoprotein ratios or protein levels relative to vehicle-treated cells (CON group). For the experimental groups, cells were treated with 100 ng/mL TNFα (T group), 100 ng/mL TNFα + 10^6^ CFU/mL HY7302 (T + 10^6^ group), or 100 ng/mL TNFα + 10^7^ CFU/mL HY7302 (T + 10^7^ group). Data are expressed as the mean ± SD (*n* = 4). # *p* < 0.05 and ### *p* < 0.001 compared with C group. * *p* < 0.05 and ** *p* < 0.01 compared with DE group. Protein values with different letters are significantly different; *p* < 0.05 (a > b > c).

CMT93 colon epithelial cells were incubated with different concentrations of HY7302 for 24 h to determine the effect on cell survival. A Cell Counting Kit-8 (CCK8) assay revealed that cell viability was >90% for all tested HY7302 concentrations, and only the highest concentration, 1 × 10^9^ CFU/mL, had significant toxic effects (Figure 6A). The treatment of CMT93 cells with 1 × 10^6^ CFU/mL HY7302, increased the mRNA levels of the tight junction components TJP-1 (1.25-fold) and OCLN-1 (1.46-fold), but did not affect the CLDN-4 mRNA level (Figure 6B–D). The effect of HY7302 on tight junction integrity in cells challenged with TNFα-induced inflammation (100 ng/mL) was evaluated. In cells stimulated with TNFα (TNF group), the mRNA levels of TJP-1 (0.81-fold), OCLN-1 (0.92-fold), and CLDN-4 (0.75-fold) decreased relative to the CON group (Figure 6E–G). The mRNA levels of TJP-1 and OCLN-1 were significantly increased in TNFα-stimulated cells treated with HY7302 (TNF + HY7304 group), but the CLDN-4 mRNA levels were unchanged (Figure 6E–G). Moreover, TNFα stimulation increased the production and release of pro-inflammatory cytokines and MMP-9. The mRNA levels of TNF (1.91-fold), MMP-9 (3.41-fold), and IL-6 (6.05-fold) were increased in the TNF group relative to the CON group (Figure 7A–C). Co-treatment with 1 × 10^6^ CFU/mL HY7302 (TNF + HY3702 group) attenuated up-regulation of the inflammatory factor mRNA levels (TNF 2.48-fold, MMP-9 1.12-fold, and IL-6 5.09-fold relative to the CON group, Figure 7A–C). Similarly, TNFα stimulation and HY7302 co-treatment (T + 10^6^ and T + 10^7^ groups) suppressed the p-ERK/ERK ratio, p-p38/p38 ratio, MMP-9 protein level, and IL-1β protein level relative to the untreated TNFα-stimulated cells (T group) in a dose-dependent manner (Figure 7D,E). 

## 3. Discussion

DE, a multifactorial ocular surface disease, is characterized by tear film instability that correlates with symptoms and pathologies such as blurred vision, eye pain, and disruption of the ocular surface [40]. The etiology of DE is complex, and damage caused by increased ocular surface inflammation or apoptosis and corneal and conjunctival abnormalities contribute to DE pathogenesis [41]. Ocular surface desiccation is an important DE trigger factor. Prior studies have used mice, rats, and rabbits as animal models to investigate the disease mechanisms of DE. In the BAC model, the ocular preservative BAC is administered to the ocular surface twice daily for 14 days. In the atropine model, DE is induced by the administration of 1% atropine sulfate to the ocular surface three times daily for 5 days. Mouse models of Sjὃgren syndrome are used to study spontaneous inflammatory DE. Desiccating stress, in which eyes are exposed to a constant low-humidity air flow for 4 h daily, can be used to induce DE. Aging animal models are also used to study DE [42]. In addition, the severity of DE in experimental animal studies is evaluated with DE tests, including the Schirmer tear test, corneal fluorescein staining, rose bengal staining, and corneal sensitivity measured by esthesiometry.

In a prior study [37], we determined the effects of the *Limosilactobacillus fermentum* strain HY7302 in a DE mouse model. We identified that oral administration of 1 × 10^9^ CFU/kg/day HY7302 improved the CFS score and TV. The effects of HY7302 on inflammatory signaling pathways in ocular tissue in BAC-treated mice with cornea damage has not been investigated. It is very important to assess the correlation of DE severity with potential changes in the gut microbiome mediated by HY7302 administration, with the analysis of affected signaling pathways in corneal tissue. Therefore, in the present study, we aimed to investigate the association between microbiome changes and the severity of DE parameters correlated with inflammation, and to delineate the physiological and molecular mechanisms for HY3702 alleviation of DE.

In the present study, DE was induced with daily exposure to 0.1% BAC for 14 days. The efficacy of *L. fermentum* HY7302 at low and high doses (1 × 10^8^ CFU/kg/day and 1 × 10^9^ CFU/kg/day) was evaluated. As shown in our data, BAC-induced DE significantly decreased tear secretion, as measured by the TV. Furthermore, DE increased the CFS score and decreased the TBUT. Treatment with HY7302 probiotics alleviated BAC-induced DE. Corneal epitheliums were dramatically detached in the BAC-induced DE group, while oral administration of HY7302 or omega-3, used as a positive control, significantly alleviated detachment of the corneal epithelium. Together, these findings suggest that HY7302 probiotics significantly improved the phenotypes of BAC-induced DE.

Impaired tear production function in DE is related to environmental stressors of the ocular surface, which eventually cause chronic corneal epithelial damage and inflammation [43]. Inflammation contributes significantly to chronic ocular surface damage, and can impair tear film homeostasis, perpetuating a vicious cycle [44,45]. Destruction of the eye barrier by corneal dryness induces an inflammatory response to external pathogens and increases the production of inflammatory cytokines, including TNF, IL-6, IL-8, and chemokines [26]. In the present study, the p-JNK/JNK ratio and IL-1β protein level were significantly increased in the conjunctiva of the DE group relative to the CON group, as measured by Western blotting. However, these changes to the phosphoprotein ratios and protein levels were alleviated in HY7302-treated groups relative to the DE group. Contrastingly, no differences in the p-JNK/JNK ratio or IL-1β level were detected in the omega-3 positive control group relative to the DE group. The effect of HY7302 on MMP-9, which disrupts the corneal epithelial barrier function, was also investigated, identifying that HY7302 treatment significantly decreased serum MMP-9 levels and the protein MMP-9 level in DE mice ocular tissues. Interestingly, according to our prior study [37], the MMP-9 protein level of corneal tissue significantly correlates with disease severity in BAC-induced DE. Consistently, in the present study, BAC-induced DE increased serum IL-20 levels, which was alleviated by HY7302 treatment but was not significantly different. Therefore, our findings suggest that HY7302 probiotics inhibit the systemic inflammatory response in tissue by regulating the expression of MMP-9 and pro-inflammatory cytokines.

Importantly, DE is often accompanied by asymptomatic epithelial disease and abnormal inflammatory responses in the cornea and conjunctiva, which could be related to increased apoptosis in ocular tissues [46]. In Sjὃgren syndrome, a systemic inflammatory disease associated with DE, pathological cell death is an important disease mechanism [47]. Apoptosis is induced by activation of caspases, such as caspase-3, and is a normal homeostatic process that functions as a defense mechanism when cells are exposed to diverse noxious stimuli [48]. We identified that the protein levels of apoptotic factors were affected in BAC-induced DE mice, with increased BAX and cleaved caspase-3 and decreased Bcl-2. HY7302 treatment increased Bcl-2 levels and decreased BAX and cleaved caspase-3 levels, suggesting decreased apoptosis. Interestingly, low-dose HY7302 treatment decreased the BAX levels in DE mice more significantly than high-dose HY7302 treatment. Thus, HY7302 intake could be more likely to have therapeutic effects in forms of DE associated with ocular epithelial inflammation and apoptosis.

Healthy aging is associated with changes to the gut–eye axis. Since the ocular surface environment and intestines are primary interfaces with the external environment, maintenance of inflammatory homeostasis in both organs is important for ocular function health related to tear secretion. The relationship between the gut microbiota and eye health has been underscored in recent findings. For example, studies on the gut–eye axis have demonstrated that the microbiota affects the pathogenesis of multiple eye diseases, including DE, age-related ocular disease, uveitis, and glaucoma [49,50]. Therefore, understanding the gut–ocular axis and role of the microbiome in eye disease is important for the development of new therapeutic approaches related to the ingestion of functional probiotics to control the microbiome. Therefore, in the present study, we evaluated the effect of HY7302 intake on gut microbial communities by analyzing gut microbiota profiles. Alpha diversity, which represents changes to diversity in experimental groups, was assessed by determining the observed number of ASVs (observed features). Also, beta diversity, which measures changes in the diversity between groups, was determined by calculating the unweighted UniFrac distance to examine differences in the microbial community composition and structure. HY7302 modestly increased gut microbiota alpha diversity, but this change was not statistically significant (Supplementary Materials, Figure S1). However, the beta diversity index significantly decreased between the CON and DE groups. Further, the beta diversity index significantly increased in HY7302-treated groups relative to the DE group, suggesting an improvement in gut microbiota diversity compared with the DE group. In addition, genus-level changes in the microbiomes of BAC-induced DE mice were partially reversed by HY7302 administration. The abundance of the family Lachnospiraceae and the family Muribaculaceae increased, while Oscillibacter decreased in HY7302-treated DE mice relative to untreated DE mice. Lachnospiraceae, Muribaculaceae, and Oscillibacter are the core of the gut microbiota community and influence the overall health of the host. These populations affect immune system regulation and the natural defenses against external infection [51].

In a recent study, gut microbiome analysis revealed compositional changes in Sjὃgren’s syndrome, which is associated with decreased tear secretion and TBUT. The abundance of the genus Bifidobacterium is significantly decreased by Sjὃgren’s syndrome, and the overall beta diversity is decreased in Sjὃgren’s syndrome, which is correlated with relative DE severity [52]. Likewise, in the present study, in the species-level analyses, the relative abundance of *Clostridium leptum* and *Bacteroides caccae* were significantly decreased in the DE group relative to the CON group (*p* = 0.002 and *p* = 0.010, respectively). However, these populations were significantly increased in HY7302H-treated DE mice relative to untreated DE mice (*p* = 0.009 and *p* = 0.036, respectively). Recent studies have identified that Bacteroides spp. comprise a major fraction of the gut bacteriome and are important for maintenance of the gut microbial food web [53]. However, changes to normal dietary probiotics could induce hyperproliferation of *Bacteroides* spp., which could cause microbiome changes induced by other intestinal symbionts. Bacteroides caccae promotes mucus degradation, which reduces intestinal inflammation by decreasing bacterial interactions with epithelial cells in the large intestine. In addition, Colidextribacter abundance is significantly correlated with pro-inflammatory metabolites generated by the gut microbiome, suggesting that Colidextribacter could produce inflammatory metabolites [54]. We also identified that *Bifidobacterium pseudolongum* abundance was significantly higher in the HY7302H-treated DE group than in the untreated DE group (*p* = 0.033). A recent study reported that *B. pseudolongum* is closely related to intestinal barrier enhancement, inflammation regulation, oxidative stress, and tight junction protein levels, in this context [55]. This suggests that in the present study, HY7302 treatment could have normalized the intestinal inflammatory response by increasing *B. pseudolongum* abundance. Together, the microbiome analyses demonstrated that HY7302 could alleviate BAC-induced DE pathologies in ocular tissue by increasing the abundances of species associated with inflammation or other chronic ocular diseases. Future studies will aim to determine the physiological effects and metabolic profile of *L. fermentum* HY7302.

Recent studies have identified that inflammatory bowel disease (IBD), a common chronic intestinal inflammatory disease, is associated with DE. IBD has detrimental effects on extraintestinal systems, such as the eye, due to intestinal wall damage [37]. A significant relationship between IBD, ocular surface damage, and recurrent corneal erosion was also identified. Regarding this, finally, in relation to the pathological microbiota mechanisms of DE and the immunomodulatory effects of HY7302 probiotics, we determined whether HY7302 could improve tight junction function in the CMT-93 mouse colon epithelial cell line. Tight junction protein 1 (TJP-1), occludin-1 (OCLN-1), and claudin-4 (CLDN-4) maintain the barrier function by regulating the permeability of intestinal epithelial cells. Further, pro-inflammatory cytokines, such as IL-1β and IL-6, are released when tight junctions are damaged and permeability increases. The pro-inflammatory factor TNFα is an important regulator of the inflammatory process in this context and affects MMP-9 production. We identified that the treatment of CMT-93 cells with HY7302 increased the mRNA levels of *TJP-1* and *OCLN-1*, but did not affect *CLDN-4* mRNA levels. HY7302 treatment also increased *TJP-1* and *OCLN-1* mRNA levels in cells stimulated with TNFα. Also, HY7302 suppressed the inflammatory response by decreasing the *TNF*, *MMP-9*, and *IL-6* mRNA levels, the p-ERK/ERK and p-p38/p38 ratios, and the MMP-9 and IL-1 β protein levels in cells stimulated with TNFα. This suggests that HY7302 ingestion could alleviate the intestinal inflammatory response by increasing tight junction protein expression, which could indirectly decrease ocular tissue inflammation.

In conclusion, the oral intake of *L. fermentum* HY7302 probiotics alleviated BAC-induced cornea damage. The TV and TBUT increased, while the CFS scores and corneal detachment injury index decreased, in DE mice treated with HY7302 relative to untreated DE mice. Moreover, HY7302 decreased DE-induced ocular inflammation and apoptosis and alleviated corneal epithelial detachment in this context. HY7302 treatment decreased inflammatory cytokine production and MMP-9 secretion in ocular tissue, which are important DE regulators. Further, HY732 increased microbiota beta diversity and altered the microbiome composition in the context of DE. Taken together, these findings suggest HY7302 could alleviate DE by regulating gut–eye axis communication via the inflammatory response. Therefore, HY7302 probiotics could potentially improve eye health by controlling intestinal health and immune regulation.

## 4. Materials and Methods

### 4.1. Preparation of L. fermentum HY7302

Preparation of *L. fermentum* HY7302 was performed, with slight modifications to the previous study method. Briefly, HY7302 was cultured in the de Man, Rogosa, and Sharpe (MRS) medium (KisanBio, Seoul, Republic of Korea) at 37 °C for 18 h to 20 h. Thereafter, the cultured cells were centrifuged at 2000× *g* for 15 min at 4 °C, washed twice with saline (0.9% NaCl), and the pellet was suspended in phosphate-buffered saline (PBS). The HY7302 suspension was freeze dried for 36 h and then spread onto an MRS plate to measure the number of viable bacteria. Afterwards, the HY7302 powder was stored at −80 °C until the in vivo and in vitro studies.

### 4.2. Animal Study

Six-week-old male Balb/c mice (ORIENT, Seongnam-si, Republic of Korea) were used in the study. All in vivo studies were approved by the Institutional Animal Care and Use Committee (IACUC, number P235002) of NDIC Co., Ltd., in Gyeonggi-do, Republic of Korea, and adhered to the guidelines on the code of practice for the housing and care of animals used in scientific procedures. Prior to the experiments, the mice were acclimatized for 1 week, with ad libitum access to water and food, on a 12 h light/12 h dark cycle. The room temperature was 23 ± 2 °C and relative humidity was 40–70%. The mice were randomly allocated to one of five groups: CON, non-DE control group; DE, topical 0.1% BAC; DE + omega-3, topical 0.1% BAC + oral omega-3 (400 mg/kg/day); DE + HY7302L, topical 0.1% BAC + oral HY7302 (10^8^ CFU/kg/day); DE + HY7302H, topical 0.1% BAC + oral HY7302 (10^9^ CFU/kg/day) (*n* = 8 mice/group). The oral administration samples were prepared by suspending HY7302 probiotic powder (1 × 10^10^ CFU/g stock powder) and the control substance (omega-3) liquid in 0.5% sodium carboxymethyl cellulose (CMC) aqueous solution. To induce DE, mouse eyes were exposed to 0.1% BAC (Sigma-Aldrich, St. Louis, MO, USA) dissolved in phosphate-buffered saline, with 5 μL/eye applied twice daily to the ocular surface for 14 days. During the same period, HY7302 (1 × 10^8^ CFU/kg/day or 1 × 10^9^ CFU/kg/day) dissolved in 0.5% aqueous carboxymethylcellulose solution was administered orally. Omega-3 fatty acids in 0.5% aqueous carboxymethylcellulose solution was orally gavaged (400 mg/kg/day) to the positive control group. 

### 4.3. Blood Parameter Analysis

Blood was collected via the abdominal vein immediately after euthanasia, and serum was prepared by centrifugation at 3000 RPM for 20 min. The serum concentrations of MMP-9 (ab253227) and IL-20 (ab235645) were measured using commercial assay kits (Abcam, Cambridge, UK).

### 4.4. Measurement of Tear Volume, Corneal Fluorescein Score, and Tear Break-Up Time

The tear volume was measured in each mouse, following an abdominal injection of 10 mg/kg xylazine/100 mg/kg ketamine. The tear amounts were measured using Schirmer’s test strips (Bio Color Tear Test, Bio Optics, Seongnam-si, Republic of Korea). The tear break-up time (TBUT) was determined using a cobalt blue slit lamp after the corneas were treated with 0.5% fluorescein solution on day 14 of BAC treatment. The time (sec) until the appearance of the first crack line on the dried tear film layer was recorded. Subsequently, the corneas were washed by an atropine antidote treatment (Alcon, Seoul, Republic of Korea). The fluorescein sodium solution (0.1%) was applied to the cornea, and corneal images were captured under blue light (Micron-IV, Phoenix, Kawasaki, Japan). The corneal fluorescein staining (CFS) score was calculated using a 0–3 point scale. The cornea was divided into five areas, which were scored individually, and the values were added to obtain the combined score.

### 4.5. Histological Analysis

The eyes were fixed with 4% paraformaldehyde and embedded in paraffin. Sections were obtained, stained with hematoxylin and eosin (H&E), and analyzed using light microscopy (Nikon Eclipse E600 microscope, Nikon Corporation, Tokyo, Japan). The number of corneal epithelium detachments per area was calculated using Image J software (version 1.54i).

### 4.6. Western Blotting

Mice corneal tissue or cells were lysed using pro-prep buffer (iNtRON Biotechnology Inc., Seoul, Republic of Korea), containing proteinase and phosphatase inhibitors. The homogenates were centrifuged at 10,000× *g* for 15 min at 4 °C and the supernatants were collected. The total protein concentration was measured using a Bio-Rad protein assay kit (Bio-Rad, Hercules, CA, USA). The protein lysates (18 µg) were separated on 4–15% precast gradient SDS-PAGE gels and transferred to PVDF membranes. The membranes were incubated at 4 °C overnight with primary antibodies in Tris-buffered saline, containing 0.05% Tween-20 (TBS-T) and 5% skim milk. After washing with TBS-T, the membranes were incubated in 5% non-fat dried milk, containing a secondary antibody conjugated to IgG horseradish peroxidase for 1 h. The protein bands were visualized using an EZ-Western Lumi Femto kit (DoGenBio, Seoul, Republic of Korea) and a LAS-4000 imager (GE Healthcare Life Sciences, Marlborough, MA, USA), which was also used to quantify band density. B-cell lymphoma 2 (Bcl-2 D17C4, cs3498), Bcl-2-associated X protein (BAX, cs2772), cleaved caspase-3 (cleaved Cas-3 Asp175, 5A1E, cs9664), glyceraldehyde 3-phosphate dehydrogenase (GAPDH 14C10, cs2118), phospho-p44/42 MAPK (p-ERK Erk1/2 137F5, cs4695), p44/42 MAPK (ERK Erk1/2 Thr202/Tyr204, cs4370), phospho-c-Jun N-terminal kinases (p-JNK Thr183/Tyr185 81E11, cs4668), c-Jun N-terminal kinases (JNK, cs9252), Interleukin 1 beta (IL-1β D3H1Z, cs12507), matrix metalloproteinase-9 (MMP-9 E7N3Y, cs24317), and anti-rabbit IgG HRP-linked secondary antibodies were purchased from Cell Signaling (Cell Signaling Technology, Danvers, MA, USA).

### 4.7. Microbiome 16s rRNA Gene Amplification and Sequencing

The sequencing libraries with amplified V3-V4 regions were used, according to the Illumina 16s Metagenomic Sequencing Library Preparation Guide (Illumina, San Diego, CA, USA). The input gDNA (2 ng) was PCR amplified with 5× reaction buffer, 1 mM dNTP mix, 500 nM of each universal F/R PCR primer, and Herculase II fusion DNA polymerase (Agilent Technologies, Santa Clara, CA, USA). The thermal cycling for the first PCR step included 3 min denaturation at 95 °C, 25 cycles of 30 s at 55 °C and 30 s at 72 °C, followed by a 5 min final extension at 72 °C. For sequencing, the V3-V4 regions of the bacterial 16s rRNA gene were amplified using primer set 341F (5′-TCGTC GGCAGCGTCAGATGTGTATAAGAGACAGCCTACGGGNGGCWGCAG-3′) and 806R (5′-GTCTCGTGGGCTCGGAGATGTGTATAAGAGACAGGACTACHVGGGTATCTAATCC-3′). The PCR products were purified using AMPure beads (Agencourt Bioscience, Beverly, MA, USA). Following purification, 2 µL of the PCR product from the first step was PCR amplified for final library construction using the Nextera XT Indexed Primer (Illumina, San Diego, CA, USA). The thermal cycling in the second PCR step was performed as described for the first step but with 10 cycles. The qPCR was conducted according to the qPCR Quantification Protocol Guide (KAPA Library Quantification kits for Illumina Sequencing platforms) and quantified using TapeStation D1000 ScreenTape (Agilent Technologies, Waldbronn, Germany). All datasets have been deposited in the NCBI Gene Expression Omnibus, under accession number PRJNA1065618.

### 4.8. Microbiome Bioinformatic Analysis of 16s rRNA Sequencing Data

The 16s rRNA amplicon sequence data were analyzed using the QIIME2 platform (version 2023.9, access date: 25 August 2023) [56]. The sequences were demultiplexed using the q2-demux plugin and the sequences with low quality scores were removed using the DADA2 (version 1.15.0), generating an amplicon sequence variants (ASVs) table [57]. The ASVs were aligned using the MAFFT (version 7.490) and were used to generate a rooted phylogenetic tree for phylogenetic diversity analysis, using FastTree 2 (version 2023.9) [58,59]. Taxonomy analysis was performed using the QIIME2 feature classifier (version 2023.9), with a 99% identity threshold in regard to the Silva 138 database [60,61]. The alpha diversity metrics of the observed features were calculated to measure microbial diversity. A non-parametric Kruskal–Wallis test was used to determine the statistical significance of the differences in microbial diversity. Unweighted UniFrac distance metrics were analyzed using principal coordinate analysis (PCoA) [62,63], and the statistical significance of the differences in the PCoA plots between the groups was assessed using permutational multivariate analysis of variance (PERMANOVA). Furthermore, using the linear discriminant analysis effect size (LEfSe) method, significant differences in the relative abundance of the bacterial composition between the HY7302 low and high groups and DE groups were identified (LDA > 3.0) (Supplementary Materials, Figure S2). Correlations between gut microbiota and the DE parameters (MMP9 IL-20, TV, TBUT, and detachment) were calculated by using Spearman’s rank correlation coefficient in the R software package (Version 3.6.6.).

### 4.9. Cell Culture

The mouse CMT-93 (CCL-223) colon epithelial cell line was purchased from ATCC (Manassas, VA, USA). CMT-93 cells were cultured in DMEM/F12, containing 10% FBS and 1% penicillin/streptomycin (P/S), in a humidified 5% CO_2_ incubator at 37 °C. The HY7302 probiotics were prepared as a 1 × 10^10^ CFU/mL stock solution in distilled water and then diluted in a medium to achieve final concentrations of 1 × 10^6^ CFU/mL or 1 × 10^7^ CFU/mL. The cells were treated with 100 ng/mL TNFα to increase epithelial tight junction permeability.

### 4.10. RNA Isolation and Quantitative Polymerase Chain Reaction (q-PCR) Analysis

The RNA was extracted using TRIzol and a total of 2 μg RNA was reverse transcribed into cDNA using a commercial kit (Maxime RT PreMix Kit, iNtRon Seongnam, Republic of Korea). The cDNA was analyzed via qPCR (Applied Biosystems, Carlsbad, CA, USA), using the TaqMan probe-based gene expression analysis system, in combination with the TaqMan gene expression master mix (Applied Biosystems, Waltham, MA, USA). *Tight junction protein-1* (*TJP-1*, Mm01320638_m1), *tight junction protein-2* (*TJP-2*, Hs00910543_m1), *occludin-1* (*OCLN-1*, Mm00500910_m1), *claudin-4* (CLDN-4, Mm00515514_s1), *tumor necrosis factor* (*TNF*, Mm00443258_m1), *MMP-9* (Mm00442991_m1), and *Interleukin-6* (*IL-6*, Mm00446190_m1) transcripts were quantified using gene-specific primers. The target gene mRNA levels were normalized against the corresponding level of *GAPDH* mRNA (Mm99999915_g1). To compare the gene expression levels between the groups, the relative mRNA levels were calculated using the 2^ΔΔCT^ method.

### 4.11. Statistical Analyses

Animal mRNA and protein data are expressed as mean ± standard deviation (SD). The data were analyzed using a one-way ANOVA and Duncan’s test (SPSS, version 6.0; Chicago, IL, USA). Statistical significance was accepted when *p* < 0.05. The cell mRNA data were analyzed and compared statistically with an unpaired two-tailed Student’s *t*-test, using SPSS version 26.0 (IBM, Somers, NY, USA). The cell viability and proteins were analyzed using a one-way ANOVA and Duncan’s test (SPSS, version 6.0; Chicago, IL, USA). Statistical significance was accepted when *p* < 0.05.

## Data Availability

Data is contained within the article and Supplementary Materials.

## Supplementary Materials

The following supporting information can be downloaded at: https://www.mdpi.com/article/10.3390/ijms25063528/s1.
