# Supplementary material for: Consumption of Limosilactobacillus fermentum Inhibits Corneal Damage and Inflammation in Dry Eye Disease Mouse Model through Regulating the Gut Microbiome
Source: Int J Mol Sci. 2024 Mar 20;25(6):3528. doi: 10.3390/ijms25063528 (PMC10971444; doi:10.3390/ijms25063528)

International Journal of
Molecular Sciences

MDPI

Article

Consumption of *Limosilactobacillus fermentum* Inhibits Corneal Damage and Inflammation in Dry Eye Disease Mouse Model through Regulating the Gut Microbiome

Kippeum Lee, Hyeonjun Gwon, Jae Jung Shim, Joo Yun Kim * and Jae Hwan Lee

R&BD Center, Hy Co., Ltd., 22 Giheungdanji-ro 24 Beon-gil, Giheung-gu, Gyeonggi-do, Yongin-si 17086, Republic of Korea
* Correspondence: monera@hy.co.kr

Figure S1. Effect of HY7302 on the taxonomy difference genus profile in BAC-induced cornea damag mice. Taxonomic abundance comparisonat genus level from 16S rDNA sequencing. CON (Control group); DE (0.1% BAC treated group); DE + Ω-3 (0.1% BAC with 200mg/mL omega-3 treated mice); DE + HY7302L (0.1% BAC with 10^8 CFU/mL HY7302 treated mice); DE + HY7302H (0.1% BAC with 10^9 CFU/mL HY7302 treated mice).

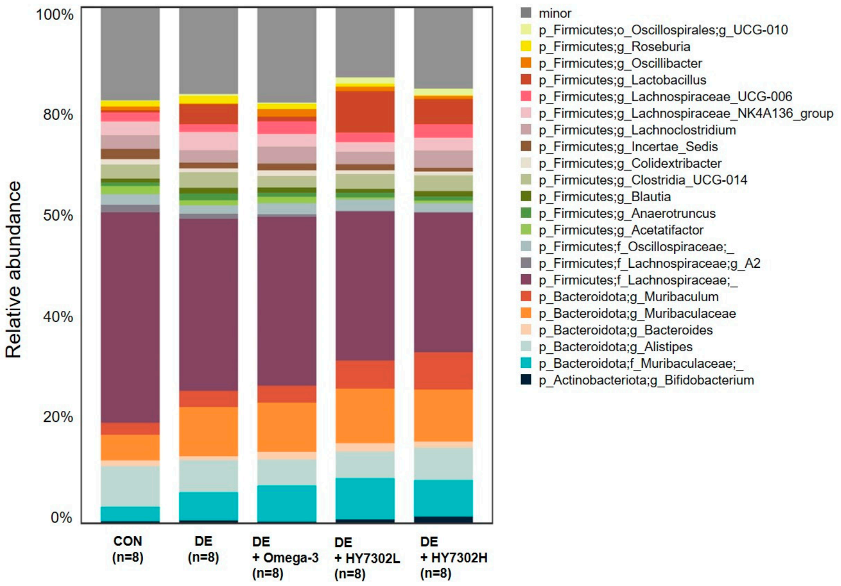

Figure S2. Effect of HY7302 on microbial taxa differing in BAC-induced cornea damag. Linear discriminant analysis effect size (LEfSe) results (A) a bar plot between DE (Dry Eye) and Control group. (B) Omega 3, (C) HY7302 L (Low concentration), and (D) HY7302 H (High concentration) compared to the DE group (LDA > 2.0)

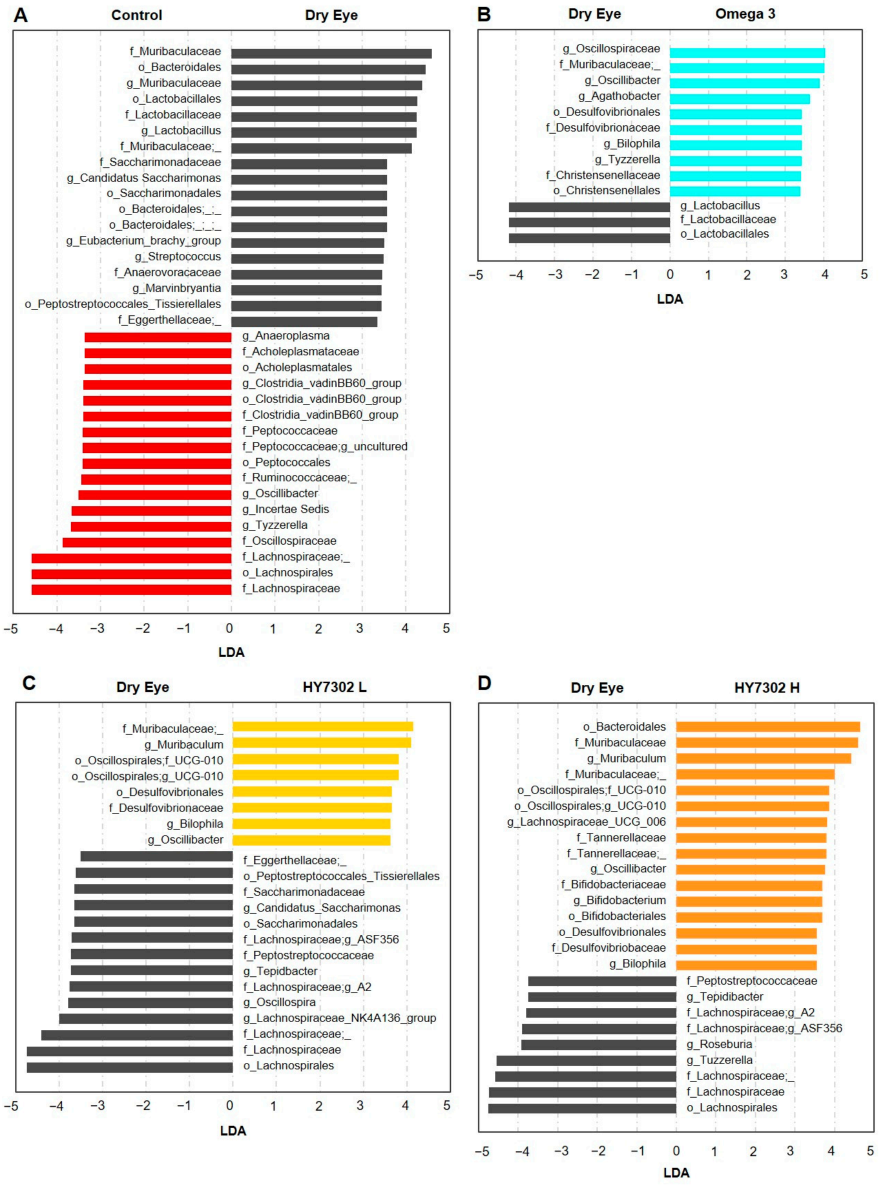

Supplement: Supplementary file 1 [file ijms-25-03528-s001.zip › ijms-2902476-supplementary.pdf]
